# A Content Analysis of Indoor Tanning Twitter Chatter During COVID-19 Shutdowns: Cross-Sectional Qualitative Study

**DOI:** 10.2196/54052

**Published:** 2024-03-04

**Authors:** Laurie Groshon, Molly E Waring, Aaron J Blashill, Kristen Dean, Sanaya Bankwalla, Lindsay Palmer, Sherry Pagoto

**Affiliations:** 1 University of Florida Gainesville, FL United States; 2 University of Connecticut Storrs, CT United States; 3 San Diego State University San Diego, CA United States; 4 UMass Chan Medical School Worcester, MA United States

**Keywords:** attitude, attitudes, content analysis, dermatology, opinion, opinion, perception, perceptions, perspective, perspectives, sentiment, skin, social media, social media, sun, tan, tanner, tanners, tanning, tweet, tweets, Twitter

## Abstract

**Background:**

Indoor tanning is a preventable risk factor for skin cancer. Statewide shutdowns during the COVID-19 pandemic resulted in temporary closures of tanning businesses. Little is known about how tanners reacted to losing access to tanning businesses.

**Objective:**

This study aimed to analyze Twitter (subsequently rebranded as X) chatter about indoor tanning during the statewide pandemic shutdowns.

**Methods:**

We collected tweets from March 15 to April 30, 2020, and performed a directed content analysis of a random sample of 20% (1165/5811) of tweets from each week. The 2 coders independently rated themes (κ=0.67-1.0; 94%-100% agreement).

**Results:**

About half (589/1165, 50.6%) of tweets were by people unlikely to indoor tan, and most of these mocked tanners or the act of tanning (562/589, 94.9%). A total of 34% (402/1165) of tweets were posted by users likely to indoor tan, and most of these (260/402, 64.7%) mentioned missing tanning beds, often citing appearance- or mood-related reasons or withdrawal. Some tweets by tanners expressed a desire to purchase or use home tanning beds (90/402, 22%), while only 3.9% (16/402) mentioned tanning alternatives (eg, self-tanner). Very few tweets (29/1165, 2.5%) were public health messages about the dangers of indoor tanning.

**Conclusions:**

Findings revealed that during statewide shutdowns, half of the tweets about indoor tanning were mocking tanning bed users and the tanned look, while about one-third were indoor tanners reacting to their inability to access tanning beds. Future work is needed to understand emerging trends in tanning post pandemic.

## Introduction

In the United States, 1 in 5 people will develop skin cancer in their lifetime [1]. Melanoma, the deadliest type of skin cancer, is the most common cancer among young adults aged 25-29 years [2]. Excessive exposure to UV radiation from either the sun or artificial sources (eg, tanning beds) is a major risk factor for skin cancer [3]. On March 11, 2020, the World Health Organization declared COVID-19 a pandemic, and states across the United States enforced stay-at-home orders, forcing businesses to close their doors. The shutdowns in the United States served as a natural experiment of the impact of tanning businesses closing on indoor tanners, as demand for tanning services tends to peak between January and June, coinciding with the COVID-19 2020 shutdowns [4]. Twitter (subsequently rebranded as X) data may be useful for understanding indoor tanning attitudes, given that young adults who are indoor tanning are almost twice as likely to use Twitter regularly than those who do not [5]. Another study assessed the frequency of mentions of indoor tanning on Twitter and found that in a 2-week period, 120,354 unique users made 154,486 tweets that mentioned the words indoor tanning, tanning bed, tanning booth, tanning salon, sun bed, or sun lamp, and these tweets reached 113,888,616 users [6].

Other studies have delved into the content of tweets about indoor tanning. For example, 1 study examined tweets that contained the phrases “tanning bed” or “tanning salon” and found that most tweets (71.2%) were posted by tanners and either expressed positive sentiment about indoor tanning, negative tanning bed experiences, or tanning-related injuries [7]. Another study of tweets containing keywords for tanning bed use and burning revealed that in 2013, over 15,000 had these keywords, and 64% described a tanning bed–induced burn [8]. Together, these studies reveal that Twitter may provide insights into tanners’ attitudes and behaviors.

This study aimed to examine Twitter chatter about indoor tanning during the COVID-19 shutdowns (March 15 to April 30, 2020). Stay-at-home orders became colloquially known by several terms, such as “shutdowns” and “lockdowns,” but all terms refer to the orders issued by local and state officials that limited business activities to those deemed essential (eg, grocery stores, pharmacies, and hospitals) and limited residents’ “nonessential” travel outside of the home [9]. The majority of stay-at-home orders (eg, shutdowns) began in March 2020, and by March 31, 2020, a total of 42 states and US territories had issued stay-at-home orders, affecting 73% of all US counties [10,11]. Location data from mobile devices suggest that compliance with restrictions was high, with 97.6% of counties with mandatory stay-at-home orders reporting a decrease in median population movement immediately after the start dates of the stay-at-home orders [10]. We were interested in whether tanners found alternative means of accessing tanning beds if they discussed interest in UV tanning alternatives (eg, sunless tanners), and their reactions to having no access to commercial tanning beds. Given the proliferation of misinformation about the impact of UV radiation on COVID-19 that appeared to have begun after former US President Donald Trump proposed the idea that UV light could be used inside the body to remedy COVID-19 [12], we also examined the presence of misinformation in tweets about tanning beds [13,14].

## Methods

### Overview

**Figure 1:**
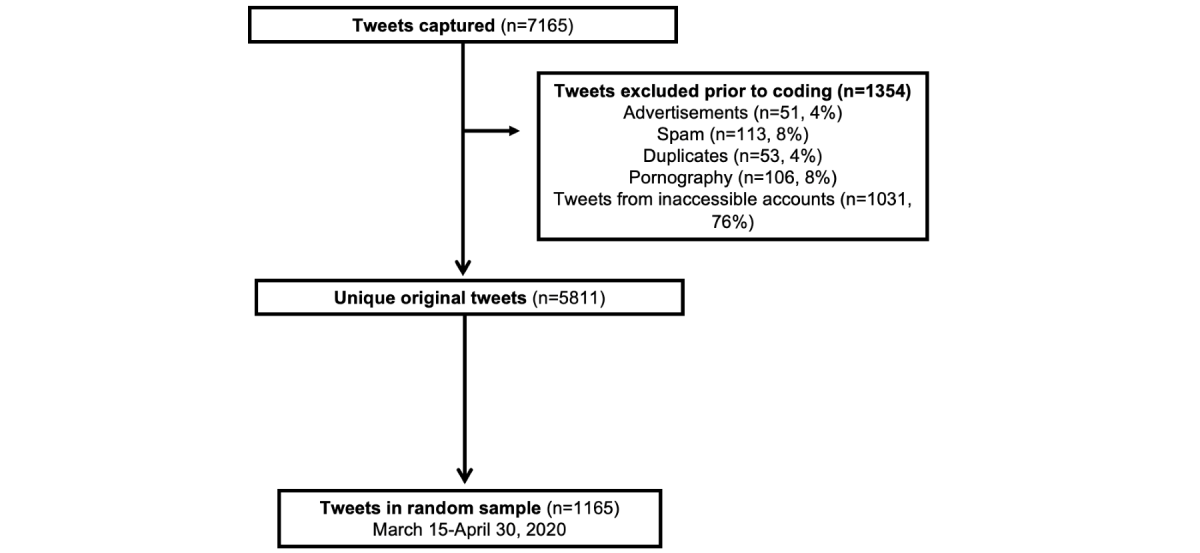
Tweet sampling and the construction of the analytic sample.

This was a cross-sectional qualitative study of public tweets about indoor tanning during the COVID-19–related shutdowns. We searched Twitter for 2 common lay terms, “tanning bed” and “tanning salon,” that refer to “indoor tanning,” a public health term that refers to tanning using artificial UV light–producing devices [7,15]. Using the R package (R Foundation for Statistical Computing) *rtweet*, we captured tweets that occurred between March 15, 2020, one of the first days of the COVID-19 statewide business shutdowns, and April 30, 2020 [16,17]. We excluded retweets because our interest was in the original thoughts of users, but we included “quote tweets,” which contain the tweeter’s own sentiments. We removed tweets that were advertisements, pornography, or from accounts that became private or were suspended between the data capture and the qualitative coding process in April 2021 (Figure 1). Of the 5811 tweets captured, we randomly sampled 20% (n=1165) of eligible tweets captured per week during the sampling window to capture conversation from the entire sampling window, consistent with other studies of tweets [18]. Table 1 contains paraphrased tweets to protect the privacy of the users.

**Table 1:** Topics of tweets (n=1165) about indoor tanning on Twitter during statewide shutdowns (March 15 to April 30, 2020), by user type. Tweets could be coded in more than 1 tweet category.

Tweet category by user type				Tweets, n (%)		Illustrative examples^a^
**People who likely do not tan indoors (n=589 tweets)**						
	Mocking tanners, tan people, or the act of tanning			562 (95.4)		Some people are about to meet their real girlfriends for the first time with the tanning bed closed hahaha.
	Mocking tweets mentioning Donald Trump			448 (76.1)		Trump went in the tanning bed too long. Looks like a burnt Cheeto.
	Health warnings			30 (5.1)		Sorry if you’re a person that uses the tanning bed, you are ruining your skin’s health and look! Proud to be pale and skin cancer free. I used to tan in a tanning bed, but you get older and your wrinkles hide small objects.
**People likely to tan indoors (n=402 tweets)**						
	**Missing tanning**			260 (64.7)		I need the tanning bed to reopen, being pale makes me depressed. Having serious tanning bed withdrawals, this is killing me!
		Appearance-related missing tanning	77 (30)		I need the tanning bed to open back up. I look so pale I can’t stand it.	
		Mood-related missing tanning	13 (5)		I need the tanning bed to reopen, that’s my stress reliever!	
		Withdrawal from indoor tanning	12 (4.6)		Anyone else going through tanning bed separation anxiety? This hurts	
	Expressing a desire to buy a home tanning bed, bought a tanning bed, or looking to use someone else’s home tanning bed			90 (22.4)		I will buy a tanning bed if this quarantine continues. PSA who’s got a tanning bed for me to use?! I’m desperate.
	Positive sentiment about tanning			69 (17.2)		So happy I have a tanning bed during this, I need to be tan.
	Use of alternative behaviors			15 (3.9)		Give me some recs for self-tanners since my tanning salon is closed! Ordered some self-tanner because this no tanning bed thing is killing me.
	Arguments against messages that tanning is unhealthy or presents indoor tanning misinformation			24 (6)		Let’s reopen the tanning salon, I think we can all agree that UV light will help kill the virus.
	Other			27 (6.9)		Burnt my face in the tanning bed and now I don’t look good.
**Tanning salon employees (n=4 tweets)**						
	Tanning salon employee chatter			4 (100)		Will these mandatory closing impact the tanning salon I work at?
**People whose tweets do not indicate whether they indoor tan (n=170 tweets)**						
	Unrelated to indoor tanning, tweets by people who do not indoor tan, or unclear whether the speaker is a tanner			168 (98.8)		That tanning bed scene in the final destination movie is creepy.
	Argues against messages that tanning is unhealthy or presents indoor tanning misinformation			2 (1.2)		I’m gonna open a coronavirus clinic, ordered a tanning bed and some Lysol. I’ll save everyone!

^a^While all tweets included in the analysis were posted publicly, to protect the privacy of individuals who posted these tweets, we paraphrased the words of tweets in a way that prevents the content of the tweet from being searchable without changing its meaning.

### Statistical Analysis

We conducted a directed content analysis of tweets using a codebook from our 2016 Twitter study about indoor tanning [7,19]. We modified the codebook after examining a subsample of 100 tweets. The original codebook had 9 codes: a desire to use a tanning bed, sleeping in a tanning bed, tanning-related injury, a complaint about or negative experiences tanning, tanning salon employee chatter, mocking tanners or tanning, health warnings about indoor tanning, pushback against “tanning is unhealthy” messaging or antitanning legislature, and references to indoor tanning in the context of an unrelated topic (eg, movie quote). We expanded the codebook to include 3 additional codes for tweets in which the user expressed that they missed being able to go indoor tanning, expressed a desire to buy a home tanning bed, crowdsourced followers to use a home tanning bed, and mentioned the use of UV tanning alternatives (eg, self-tanners). We eliminated 3 codes (ie, sleeping in a tanning bed, tanning-related injury, a complaint about, or negative experiences) because they were not represented within the current data set. We also coded tweets as posted by people who were likely to indoor tan (based on their admission of tanning or having tanned in their tweets), tanning salon employees (based on the content of their tweets), people who are not likely to indoor tan (based on their mocking indoor tanning or discussing the risks of indoor tanning), and people whose tweets do not indicate if they indoor tan or not. If a tweet seemed to be posted by a tanning salon employee but referred to their individual tanning behavior, we coded the tweet as being posted by someone likely to indoor tan. After finalizing the codebook, 2 coders independently coded all 1165 tweets (100% double-coded). Discrepantly coded tweets were discussed to reach a consensus.

We calculated interrater reliability and Cohen κ for each coding category. Interrater agreement of tweet categories ranged from 94% to 100%, and Cohen κ statistics ranged from 0.6654 to 1.0. Interrater agreement among coders was 94% (κ=0.9106). We summarized the proportion of tweets posted by those likely to indoor tan, tanning salon employees, those unlikely to indoor tan, and those whose tweets do not indicate whether they indoor tan. We then reported the frequency of tweet categories by user types. Analyses were conducted using SAS 9.4 (SAS Institute, Inc).

### Ethical Considerations

This study does not meet the definition of human participants research and thus did not require Institutional Review Board approval. However, to protect the privacy of users who may not expect public tweets to be used in research, we paraphrased tweets to render the tweet’s content unsearchable while preserving the meaning. We confirmed that the paraphrased content did not produce the original tweet through searches.

## Results

### Overview

In our final sample of 1165 tweets, 1144 (98%) were posted by unique Twitter accounts. A total of 93% (1084/1165) of tweets in our analytic sample were from the search term “tanning bed,” while only 7% (81/1165) were from the search term “tanning salon.”

Half of the tweets (589/1165, 50.6%) came from users unlikely to indoor tan, while 34.5% (402/1165) were posted by users who seemed likely to indoor tan (Table 1). Very few tweets (4/1165, 0.4%) appeared to be posted by tanning salon employees, and in the remaining 14.5% (170/1165) of tweets, the content did not clearly indicate whether the user was an indoor tanner.

### Tweets From People Unlikely to Indoor Tan

The majority (562/589, 95%) were classified as mocking tanners, tan people, or the act of tanning. Among these, the majority (446/589, 75.7%) mocked former US President Donald Trump’s skin tone, and 20.6% (116/589) mocked the appearance of tanners and the use of tanning beds in general. The remaining 5% (30/589) of tweets from users unlikely to be indoor tanners contained health warnings about indoor tanning.

### Tweets From People Likely to Indoor Tan

Nearly two-thirds (260/402, 64.7%) were coded as “missing tanning,” meaning the user expressed they missed tanning, their frustration that they could not go tanning, or their eagerness to get back to tanning (Table 1). Within this category, 60% (156/260) of tweets did not mention a specific reason they missed tanning, but 30% (77/260) indicated they missed indoor tanning for appearance-related reasons, 5% (13/260) indicated they missed indoor tanning for mood-related reasons, and 5% (12/260) indicated withdrawal symptoms from being restricted from indoor tanning. The second most common theme among tweets from likely tanners was general positive attitudes about indoor tanning (69/402, 17.2%), followed by the desire to buy a home tanning bed or use someone else’s (90/402, 22.3%), misinformation about tanning (24/402, 6%), and finally, use of alternative tanning methods such as self-tanner and bronzer makeup (16/402, 3.9%; Table 1).

### Other Tweets

The content of the remaining tweets (170/174, 98.8%) made it unclear whether the user was an indoor tanner. The vast majority (165/170, 95.9%) mentioned tanning beds in the context of an unrelated topic (eg, movie scene). Tweets posted by tanning salon employees (n=4) were rare and included observations of occurrences in the workplace.

## Discussion

### Overview

About half of the tweets (589/1165, 50.6%) using the keywords “tanning bed” or “tanning salon” during the COVID-19 pandemic shutdowns in March and April 2020 were not by people who likely use tanning beds. Most of these tweets were mocking people who tan, tanning beds, or the tanned look. The next largest set of tweets (402/1165, 34.5%) seemed to be by people who use tanning beds, as evidenced by their content, which focused on lamenting the inability to tan during the shutdown, expressing the desire for a home tanning bed, expressing positive sentiment about tanning beds, discussing alternative ways to get a tan in the absence of tanning beds, or promoting misinformation.

The finding that only about one-third of tweets (402/1165, 34.5%) appeared to be from indoor tanners is in contrast to a similar investigation by Waring et al [7], where twice the proportion of tweets (699/978, 71.2%) using the same search terms in March 2016 appeared to be from indoor tanners [7]. This finding could be due to declining rates of tanning bed use in recent years [20] or that COVID-19 shutdowns curtailed indoor tanning, which may have decreased chatter about it [20]. Another possibility could be that the proportion of tanning-related tweets that were negative chatter about former US President Donald Trump’s skin color increased from 2016 to 2020 [21-25]. Waring et al [7] found only 10.7% of tweets in 2016 were mocking tanners and the tanned look, compared to 48% (562/1165) of tweets from 2020 in this study. Among tweets that mocked tanners, the vast majority (448/562, 79.7%) mocked former US President Donald Trump, accounting for 38% (446/1165) of all tweets. Criticism of a tan-appearing public figure may shift social norms about indoor tanning for the better or worse, depending on how people feel about that public figure. Perceived social norms strongly predict indoor tanning [26] and increased negative sentiment toward indoor tanning and a tan appearance may shift appearance-related social norms. Future research should explore how negative sentiment on social media about tanned celebrities influences indoor tanning behavior and attitudes.

While most posted tweets lamented the inability to tan, interestingly, very few (16/402, 3.9%) mentioned using tanning alternatives (eg, sunless tanners). Some tanners may have been more interested in gaining access to UV tanning than non-UV tanning, even though the latter was far more accessible. However, those who switched to non-UV tanning may have been less inclined to discuss this on Twitter, perhaps simply because non-UV tanning was more accessible or perhaps to the extent they felt the stigma around admitting to getting a “fake tan” [27,28]. The COVID-19–related shutdowns may have been a missed opportunity to promote sunless tanning products. Because orange-appearing skin was also the focus of tweets mocking Donald Trump (eg, “Trump been in the tanning bed too long? He looks like a Cheeto”), these tweets may have also negatively impacted social norms around sunless tanning products. Many tanners fear sunless tanning products will create an orange appearance because early products had this effect [27,28]. Future research should examine how social norms around tanning beds and sunless tanning are influenced by social media conversations.

The most common type of tweet among tanners expressed that they missed tanning and 39% (260/402) of these mentioned reasons they missed tanning. Appearance-related reasons were by far the most common (77/260, 30%). The increase in the use of videoconferencing software during the COVID-19 pandemic has been shown to have exacerbated appearance-related concerns, leading to an increase in cosmetic surgery consults [29,30]. Because physical appearance is well established to be among the most common reasons people use tanning beds [31,32], future studies should examine how the widespread use of videoconferencing has impacted tanning behavior.

Additional reasons people cited for missing indoor tanning included the positive impact they perceive tanning has on their mood or their discomfort with the negative effect they experience when they are unable to use tanning beds. Research has shown that 8% to 20% of tanners meet criteria for “tanning addiction,” indicators of which may include the experience of mood enhancement from tanning and withdrawal symptoms (eg, irritability) when they cannot tan [33-36]. Future research should explore how the shutdowns may have impacted tanning behavior among people qualifying as “tanning addicted.” The forced period of “cold turkey” could possibly have led some tanners to reduce or quit their tanning habit altogether. Alternatively, when tanning salons reopened, a disinhibition effect may have occurred, such that tanners increased their tanning beyond prepandemic levels after being involuntarily restricted.

Some tweets from tanners (90/402, 22%) expressed their interest in gaining access to a home tanning bed. Future studies should examine whether the small segment of indoor tanners (<10%) who use home beds grew following the pandemic shutdowns [37,38]. The impact of restricted or discouraged access to tanning beds has implications for legislative and public health efforts. For example, Australia banned commercial tanning services in 2016. Governments initiated buyback programs to discourage home tanning bed use in the states of Victoria and New South Wales [39,40]. Afterward, Australian consumer interest in tanning beds declined to less than one-fourth of preban seasonal peaks, but interest in sunless tanning was high [41]. While home tanning beds are still legally marketed in Australia, spray tanning remains more popular [41,42]. Therefore, buyback programs or legislation restricting the sale of home tanning beds may be necessary accompaniments to legislation restricting tanning businesses in the United States.

Unfortunately, we observed very few public health messages regarding the dangers of indoor tanning. Only 2.5% (29/1165) of tweets were of this type, which is even less than the 4.3% that was observed in the previous investigation of tanning bed chatter on Twitter [7]. To be sure, public health efforts were heavily focused on COVID-19 at this time. However, given the misinformation about UV and COVID-19 prevention [14], this would have been an important opportunity to underscore the dangers of indoor tanning. Interestingly, only 5 (0.4%) out of 1165 tweets contained misinformation. Misinformation themes included that UV radiation from tanning beds could kill COVID-19, that UV radiation from the sunbathing could kill COVID-19, and that indoor tanning is healthy to use as therapy. However, because tweets in our study must have contained the words tanning bed or tanning salon, they may not have captured the full range of misinformation circulating about UV and COVID-19.

This study has limitations. Our data capture was limited to 2 common lay terms typically used in the United States to refer to indoor tanning. We may not have captured tweets containing other terms that refer to indoor tanning or tweets about using non-UV tanning alternatives. Additionally, states started reopening at different times during the end of the sampling window, which may have impacted the types of tweets in our sample [43]. Further, we may have captured tweets that were posted by users outside of the United States. Few Twitter users activate their location data [44], so it is difficult to determine where all the tweets originated. As we only coded 1165 tweets from the nearly 5000 unique tweets captured during the sampling window, we may have missed interesting yet rare topics of conversation. Additionally, tanners who were most upset by the shutdowns may have been more likely to tweet about them. Among the 23% of adults in the United States that use Twitter, only 18% reside in rural areas [45,46], so our data may not have captured the full range of sentiment about indoor tanning in rural areas of the United States.

### Conclusion

Many indoor tanners appeared to miss indoor tanning during the pandemic shutdown, particularly due to appearance concerns, and some were seeking alternative ways to access tanning beds. We also discovered that, compared to a similar investigation 5 years ago, a much larger percentage of tweets about indoor tanning were very critical of indoor tanning [7]. The use of tanning beds or the appearance of having used them appears to be the target of insults that are often politically motivated on social media. Future research is needed to examine how the pandemic and the surrounding political climate affected tanning behavior and attitudes.

## Data Availability

The data sets generated and analyzed during this study are available from the corresponding author on reasonable request.

## References

[1] Riker AI, Zea N, Trinh T (2010). The epidemiology, prevention, and detection of melanoma. Ochsner J.

[2] Department OHU, Services H (2014). The surgeon general’s call to action to prevent skin cancer. United States Department of Health and Human Services.

[3] Narayanan DL, Saladi RN, Fox JL (2010). Ultraviolet radiation and skin cancer. Int J Dermatol.

[4] (2023). Tanning salons in the US - market size, industry analysis, trends and forecasts (2024-2029). IBISWorld.

[5] Stapleton JL, Hillhouse J, Coups EJ, Pagoto S (2016). Social media use and indoor tanning among a national sample of young adult nonHispanic White women: a cross-sectional study. J Am Acad Dermatol.

[6] Wehner MR, Chren MM, Shive ML, Resneck JS, Pagoto S, Seidenberg AB, Linos E (2014). Twitter: an opportunity for public health campaigns. Lancet.

[7] Waring ME, Baker K, Peluso A, May CN, Pagoto SL (2019). Content analysis of Twitter chatter about indoor tanning. Transl Behav Med.

[8] Seidenberg AB, Pagoto SL, Vickey TA, Linos E, Wehner MR, Costa RD, Geller AC (2016). Tanning bed burns reported on Twitter: over 15,000 in 2013. Transl Behav Med.

[9] Jacobsen GD, Jacobsen KH (2020). Statewide COVID-19 stay-at-home orders and population mobility in the United States. World Med Health Policy.

[10] Moreland A, Herlihy C, Tynan MA, Sunshine G, McCord RF, Hilton C, Poovey J, Werner AK, Jones CD, Fulmer EB, Gundlapalli AV, Strosnider H, Potvien A, García MC, Honeycutt S, Baldwin G (2020). Timing of state and territorial COVID-19 stay-at-home orders and changes in population movement - United States, March 1-May 31, 2020. MMWR Morb Mortal Wkly Rep.

[11] (2023). Coronavirus: timeline. U.S. Department of Defense.

[12] (2020). Coronavirus: Trump’s disinfectant and sunlight claims fact-checked. BBC News.

[13] Yamey G, Gonsalves G (2020). Donald Trump: a political determinant of COVID-19. BMJ.

[14] Seigel J (2020). Trump raises question of ultraviolet light and COVID-19. We ask doctors, scientists. The Seattle Times.

[15] Lichtenstein J, Sherertz EF (1985). Harmful effects of indoor tanning. Am Fam Physician.

[16] Mettler K (2020). CDC urges halting gatherings of 50 people or more. Washington Post.

[17] Kearney MW (2019). rtweet: collecting and analyzing Twitter data. J Open Source Softw.

[18] Heaivilin N, Gerbert B, Page JE, Gibbs JL (2011). Public health surveillance of dental pain via Twitter. J Dent Res.

[19] Hsieh HF, Shannon SE (2005). Three approaches to qualitative content analysis. Qual Health Res.

[20] Guy GP, Watson M, Seidenberg AB, Hartman AM, Holman DM, Perna F (2017). Trends in indoor tanning and its association with sunburn among US adults. J Am Acad Dermatol.

[21] Wade P (2019). How does trump maintain his orange glow?. Rolling Stone.

[22] Weiner Z (2018). Donald Trump reportedly uses a tanning bed every morning. Teen Vogue.

[23] Rogers K (2019). In the pale of winter, Trump’s tan remains a state secret. The New York Times.

[24] Wagtendonk AV (2020). What is up with that tan line photo of Trump?. Vox.

[25] Chait J (2018). Trump insists real photo revealing his fake tan is fake. Intelligencer.

[26] Carcioppolo N, Peng W, Lun D, Occa A (2019). Can a social norms appeal reduce indoor tanning? preliminary findings from a tailored messaging intervention. Health Educ Behav.

[27] Mahler HIM, Kulik JA, Harrell J, Correa A, Gibbons FX, Gerrard M (2005). Effects of UV photographs, photoaging information, and use of sunless tanning lotion on sun protection behaviors. Arch Dermatol.

[28] Pagoto SL, Schneider KL, Oleski J, Bodenlos JS, Ma Y (2010). The sunless study: a beach randomized trial of a skin cancer prevention intervention promoting sunless tanning. Arch Dermatol.

[29] Cristel RT, Demesh D, Dayan SH (2020). Video conferencing impact on facial appearance: looking beyond the COVID-19 pandemic. Facial Plast Surg Aesthet Med.

[30] Pikoos TD, Buzwell S, Sharp G, Rossell SL (2021). The zoom effect: exploring the impact of video calling on appearance dissatisfaction and interest in aesthetic treatment during the COVID-19 pandemic. Aesthet Surg J.

[31] Glanz K, Jordan A, Lazovich D, Bleakley A (2019). Frequent indoor tanners' beliefs about indoor tanning and cessation. Am J Health Promot.

[32] Howell AL, Paulins VA (2016). Women's motives for engaging in long-term habitual indoor tanning. J Fam Consum Sci.

[33] Toledo A, Yli-Uotila E, Kautiainen H, Pirkola S, Partonen T, Snellman E (2019). Tanning dependence and seasonal affective disorder are frequent among sunbathers but are not associated. Psychiatry Res.

[34] Diehl C, Rees J, Bohner G (2018). Predicting sexual harassment from hostile sexism and short-term mating orientation: relative strength of predictors depends on situational priming of power versus sex. Violence Against Women.

[35] Tripathi R, Bordeaux JS, Scott JF (2019). Inclusion of tanning use disorder in the DSM-V: implications for awareness, patient care and research. J Eur Acad Dermatol Venereol.

[36] Stapleton JL, Hillhouse JJ, Turrisi R, Baker K, Manne SL, Coups EJ (2016). The Behavioral Addiction Indoor Tanning Screener (BAITS): an evaluation of a brief measure of behavioral addictive symptoms. Acta Derm Venereol.

[37] Hillhouse J, Stapleton JL, Florence LC, Pagoto S (2015). Prevalence and correlates of indoor tanning in nonsalon locations among a national sample of young women. JAMA Dermatol.

[38] Nahar VK, Rosenthal M, Lemon SC, Holman DM, Watson M, Hillhouse JJ, Pagoto SL (2016). Characteristics and practices of adults who use tanning beds in private residences. JAMA Dermatol.

[39] (2013). NSW Government to buy back harmful tanning beds ahead of a ban on solariums next year. ABC News Australia.

[40] (2013). Vic state government introduces solarium ban legislation. SunSmart.

[41] Gordon LG, Sinclair C, Cleaves N, Makin JK, Rodriguez-Acevedo AJ, Green AC (2020). Consequences of banning commercial solaria in 2016 in Australia. Health Policy.

[42] Sinclair C, Cleaves N, Dunstone K, Makin J, Zouzounis S (2016). Impact of an outright ban on the availability of commercial tanning services in Victoria, Australia. Br J Dermatol.

[43] Miller H (2020). Reopening America: a state-by-state breakdown of the status of coronavirus restrictions. CNBC.

[44] Ribeiro S, Pappa GL (2017). Strategies for combining Twitter users geo-location methods. Geoinformatica.

[45] (2021). Social media fact sheet. Pew Research Center.

[46] Auxier B, Anderson M Social media use in 2021. Pew Research Center.

